# FastSurfer parcellation accuracy after lesion filling in moderate-to-severe traumatic brain injury

**DOI:** 10.3389/fneur.2025.1652385

**Published:** 2025-12-10

**Authors:** Evelyn Deutscher, Emily Dennis, Frank G. Hillary, Elisabeth A. Wilde, Carrie Esopenko, Ekaterina Dobryakova, Andrei Irimia, Ahmed M. Radwan, Phoebe Imms, Adam Clemente, Paul Beech, Alex Burmester, Karen Caeyenberghs, D. Juan F. Domínguez

**Affiliations:** 1 Cognitive Neuroscience Unit, School of Psychology, Deakin University, Burwood, VIC, Australia; 2 TBI and Concussion Center, Department of Neurology, University of Utah, Salt Lake City, UT, United States; 3 George E. Wahlen Veterans Affairs Salt Lake City Healthcare System, Salt Lake City, UT, United States; 4 Department of Psychology, Penn State University, State College, University Park, PA, United States; 5 Department of Neurology, Hershey Medical Center, Hershey, PA, United States; 6 H. Ben Taub Department of Physical Medicine and Rehabilitation, Baylor College of Medicine, Houston, TX, United States; 7 Department of Rehabilitation and Human Performance, Icahn School of Medicine at Mount Sinai, New York, NY, United States; 8 Center for Traumatic Brain Injury, Kessler Foundation, East Hanover, NJ, United States; 9 Rutgers New Jersey Medical School, Newark, NJ, United States; 10 Ethel Percy Andrus Gerontology Center, Leonard Davis School of Gerontology, University of Southern California, Los Angeles, CA, United States; 11 Alfred E. Mann Department of Biomedical Engineering, Andrew and Erna Viterbi School of Engineering, University of Southern California, Los Angeles, CA, United States; 12 Department of Quantitative and Computational Biology, Dana and David Dornsife College of Arts and Sciences, University of Southern California, Los Angeles, CA, United States; 13 Centre for Healthy Brain Aging, Institute of Psychiatry, Psychology & Neuroscience, King’s College London, London, United Kingdom; 14 Department of Imaging and Pathology, Translational MRI, KU Leuven, Leuven, Belgium; 15 School of Behavioural and Health Sciences, Faculty of Health Sciences, Australian Catholic University, Melbourne, VIC, Australia; 16 Department of Radiology and Nuclear Medicine, The Alfred Hospital, Melbourne, VIC, Australia

**Keywords:** traumatic brain injury, neuroimaging, MRI, lesion filling, lesion inpainting, parcellation, FastSurfer

## Abstract

**Objective:**

Focal lesions in T1-weighted (T1-w) magnetic resonance images (MRIs) of patients with moderate-to-severe traumatic brain injury (ms-TBI) can introduce errors during image processing. We tested whether errors in FastSurfer cortical parcellation could be reduced using lesion filling (virtual brain grafting (VBG)).

**Methods:**

T1-w MRIs from 140 healthy controls and 14 ms-TBI patients were shared within the ENIGMA TBI working group. A “ground truth” set of 140 *lesion-free* images was created by registering 10 healthy controls (HCs) onto each of 14 ms-TBI images. Masks indexing focal lesions (small [38 mm^3^] unilateral to large [164,291 mm^3^] bilateral) were projected onto *lesion-free* images, creating 140 synthetically *lesioned* images. *Lesioned* images underwent VBG filling to replace lesioned regions with simulated healthy brain tissue, creating 140 *VBG-filled* images. To calculate parcellation accuracy, paired sample *t-*tests of mean Dice similarity coefficients (DSCs) and percent volume differences (PVDs) for *lesioned* and *VBG-filled* images were compared to *lesion-free* images.

**Results:**

Parcellations from *lesioned* images (DSC M = 0.93, SD = 0.03; PVD M = −0.40, SD = 1.7) unexpectedly had significantly higher DSCs [*t*(111) = 19.5, *p <* 0.001] and lower PVDs [*t*(111) = 11.3, *p <* 0.001] than *VBG-filled* images (DSC M = 0.81, SD = 0.07; PVD M = −9.03, SD = 7.72).

**Interpretation:**

Parcellations from *lesioned* images were more accurate (than *VBG-filled* images) than *lesion-free* ground truth images. While likely due to a high frequency of smaller focal lesions in our sample, these results could suggest that FastSurfer parcellation may be robust in the presence of such lesions.

## 1 Introduction

Lesions in moderate-to-severe traumatic brain injury (ms-TBI) patients differ from other common acquired brain injuries (such as stroke, multiple sclerosis, and brain tumor) as they can be both focal and diffuse, varying in location, size, number, and laterality, extending through multiple tissue types [gray matter (GM), white matter (WM), and cerebrospinal fluid (CSF)], and can also occur in homologous regions of both hemispheres (1). The complex entanglement of diverse lesions and varied individual characteristics results in highly heterogeneous long-term outcomes for ms-TBI patients. To better understand outcomes, recent ms-TBI neuroimaging research has called for the need to sample across lesion characteristics (e.g., lesion size, type, number, and location) (36). However, this requires large datasets in order to attempt to capture the variety of lesions observed within ms-TBI (2, 3).

Recent global collaborations have dramatically increased TBI sample sizes through the establishment of large-scale multicenter studies or through aggregating existing data from independent studies. An example of the latter is the Enhancing NeuroImaging Genetics through Meta-Analysis (ENIGMA) international consortium that, by pooling worldwide magnetic resonance imaging (MRI) data, has revealed robust associations between behavioral deficits and brain alterations in large samples of TBI patients (4, 5). In the ENIGMA pediatric ms-TBI working group, a coordinated analysis of T1w MRI data reported significant associations between decreased cerebellar volume, particularly in the posterior lobe, and poorer executive function (6).

Despite these promising findings, multicenter studies and large-scale consortia in ms-TBI rely on automated neuroimaging tools (to enable the fast automated processing of large datasets). These tools assume normal brain anatomy for the input brain image, an assumption that is violated when including ms-TBI patients with heterogeneous lesions. Pre-processing of neuroimaging data often involves the use of automated tools that perform image processing steps such as brain extraction (7), normalization (8), tissue-class segmentation (9), and regional parcellation (10). The presence of large lesions, however, leads to diminished accuracy and even failure in these steps (11). TBI lesions often exhibit signal intensities similar to that of non-brain tissue, which therefore increases the likelihood of their misclassification during processes such as brain extraction (12) and automated parcellation.

FreeSurfer is an automated whole-brain parcellation (10) tool that has been widely used in previous TBI studies (13). However, FreeSurfer is susceptible to lesion-induced errors (14). Researchers investigating ms-TBI are often required to exclude affected regions (15) or exclude whole subjects in the case of major errors (16). This results in TBI samples containing less severe lesions, thereby reducing the heterogeneity present even in large datasets. Post-hoc manual correction techniques have been developed to remove *local* lesion-induced errors (misclassification of regions immediately surrounding a lesion) (17). However, these methods are not able to correct *global* lesion-induced parcellation errors (misclassification of regions distant from the lesion) (14, 18, 19).

A new parcellation tool based on an advanced deep learning architecture (a convolutional neural network, CNN), called FastSurfer, was recently been developed (20) that generates FreeSurfer-conform outputs. FastSurfer has been shown to have several advantages over FreeSurfer, such as faster processing times and increased test–retest reliability (20). FastSurfer has also been reported to exhibit higher intra-class correlation coefficients compared to FreeSurfer when assessing the reliability of brain atrophy measures in persons with multiple sclerosis (MS) (21). This finding is especially interesting given that FastSurfer still assumes normal brain images as input (17). Increasing evidence suggests that conducting lesion filling (also called image inpainting) prior to automated parcellation may reduce both local and global lesion-induced parcellation errors (22–25). Lesion filling is an image manipulation technique that commonly uses a binary mask to index the location of abnormal MRI signal intensities before replacing them with intensities that would be expected from healthy tissue at that same location. Using the filled brain image as input for parcellation enables compliance with the assumption of a brain with no lesion (18). For example, in a study of persons with MS, Battaglini et al. (22) developed a lesion filling tool capable of resolving the misclassification of damaged WM as GM, which was otherwise unable to be corrected using post-hoc masking of the lesions (22). A recent study by Fekonja et al. (26) showed that using enantiomorphic lesion filling before completing segmentation using FastSurfer resulted in more accurate cortical parcellations in patients with unilateral gliomas. This promising finding supported the rationale for testing similar approaches in patients with ms-TBI. To date, however, the majority of lesion filling tools have been designed for WM MS lesions (24, 27, 28), unilateral stroke lesions (29), unilateral brain tumors (25), or TBI-associated hydrocephaly (in a unilateral sample) (30) and are thus not appropriate for use in ms-TBI images with bilateral pathology.

Recently, Radwan et al. (25) developed Virtual Brain Grafting (VBG), a novel lesion filling tool enabling automatic detection and virtual repair of either unilaterally or bilaterally damaged brain tissue. In a cohort of unilateral glioma patients, images that underwent VBG filling resulted in FreeSurfer parcellations with better spatial alignment to a ground truth when compared to images that had not undergone VBG filling. VBG has already been utilized in stroke (31) and glioma patients (32), although, to the best of our knowledge, it has not yet been validated in patients with bilateral lesions, which commonly occur in ms-TBI patients.

In this study, we investigated the use of VBG lesion filling alongside FastSurfer to improve the accuracy of cortical parcellations in ms-TBI patients. Lesions identified on T1w MRI scans of ms-TBI patients were simulated on anatomical MRI images of healthy controls (HCs) before performing lesion filling using VBG. First, the registration of HC images onto VBG-filled TBI images generated a set of “*lesion-free*” images that could act as a ground truth. Utilizing binary lesion masks, TBI lesions were then inserted onto the *lesion-free* images to generate a set of simulated “*lesioned”* images. Finally, we used VBG to replace the lesioned voxel intensities with approximations of healthy tissue, generating a set of *‘VBG-filled’* images. Each set of three images—*lesion-free, lesioned,* and *VBG-filled* images—is identical in all regions outside of the lesion, enabling FastSurfer parcellation to be directly compared against images within each set. The differences in parcellation accuracy produced from the *lesioned* and *VBG-filled* images compared to the *lesion-free* images can be attributed to the presence of a lesion and the effect of lesion filling, respectively.

Our aims were twofold: First, as this is the first test of VBG inpainting in true discrete bilateral lesions, we conducted a visual check of the inpainting to observe the coherence of the filled regions with respect to the surrounding normal-appearing tissue. Second, we conducted a quantitative analysis whereby cortical regions of interest (ROIs) from the *lesioned* and *VBG-filled* images were compared. We calculated the spatial alignment and accuracy of volumes, with respect to the ROIs from the *lesion-free*, ground truth images. We hypothesized that the *VBG-filled* ROIs, when compared to *lesioned* image ROIs, would have higher spatial similarity and differ less in volume from the ground truth, indicating that VBG reduces the influence of lesion-induced parcellation errors.

## 2 Methods

### 2.1 Participants

From datasets shared within the ENIGMA adult and pediatric ms-TBI working groups, we selected T1w MRI scans from 140 HCs (10–60 years, M = 28.68 years, SD = 13.05 years, 70 males) and 14 TBI patients in the chronic stage of injury (> 6 months after injury) (10–60 years, M = 25.6 years, SD = 16.14 years, 7 males). TBI severity was defined using either (1) the Mayo classification system (33) or a Glasgow Coma Scale score (34) at the time of hospital admission; (2) loss of consciousness for 30 min or greater; (3) post-traumatic amnesia >24 h (35); and (4) positive findings of gross injury on MRIs as per evaluation by a neuroradiologist (PB).

**Figure 1:**
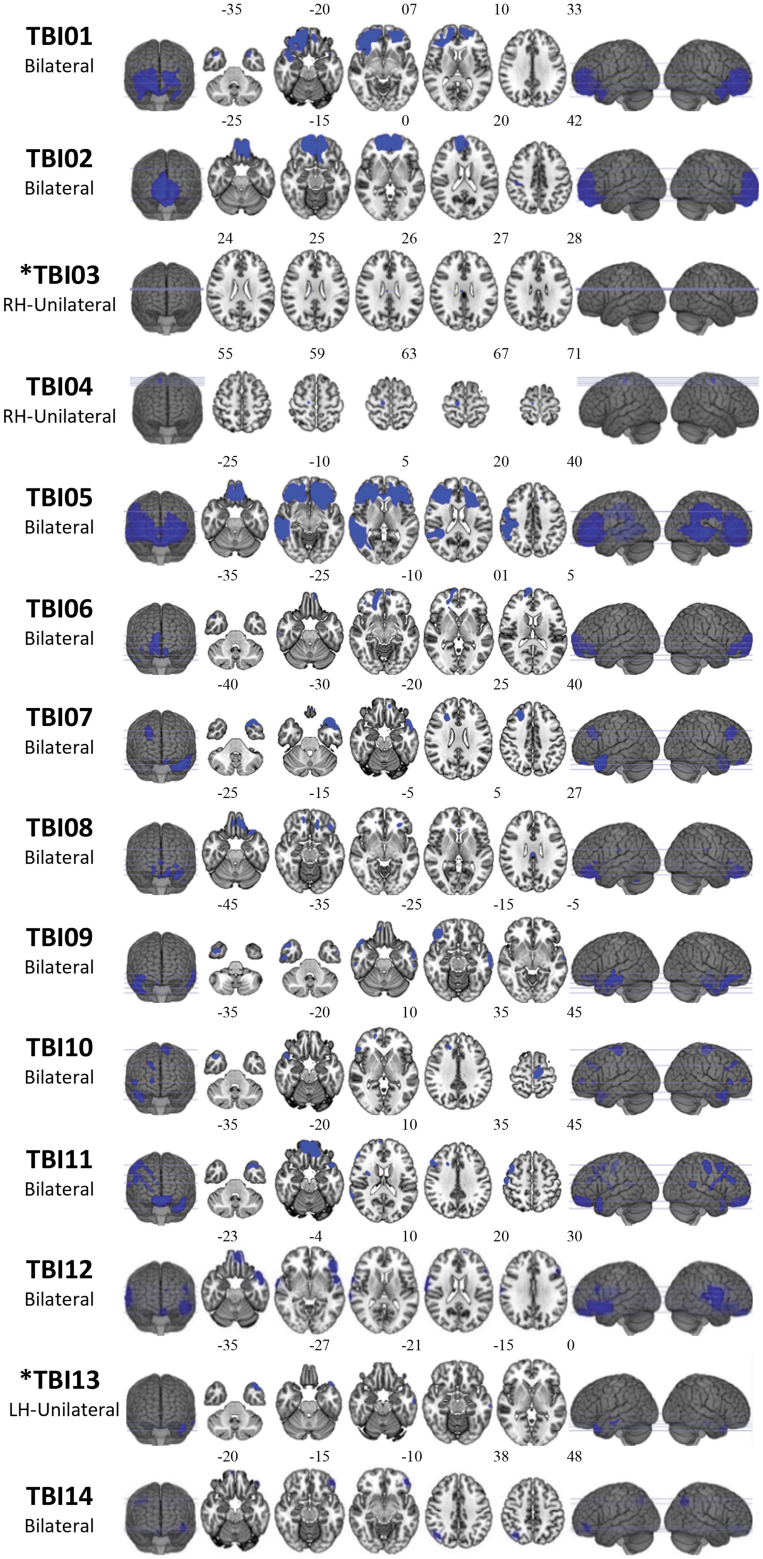
Visual depiction of lesion locations for each of the 14 individuals with ms-TBI. Lesions have been registered (using Statistical Parametric Mapping (SPM) clinical toolbox) and overlaid on top of the MNI152 template brain for visual depiction purposes. Participants who failed lesion simulation are indexed by *.

Supplementary Table 1 provides an overview of the original data collection sites. Informed consent was provided by participants in accordance with local ethics guidelines, and the privacy rights of participants have been observed, with data analysis occurring under the approval of Deakin University Ethics number 2023–267. TBI participants were excluded if they had any of the following: (1) previous TBIs; (2) previous diagnoses of neurological disorders; (3) current or previous substance abuse issues; and (4) contraindications for MRI (e.g., ferromagnetic implants). Ten HC subjects were age- and sex-matched to each TBI patient (age within 2 years; M = 0.4 years, SD = 1.13 yrs). Patients with TBI were selected to represent the heterogeneity of TBI lesions (Figure 1), including lesion size, location, and laterality (36). Specifically, 11 patients with TBI had focal bilateral lesions, two patients had focal unilateral lesions (one left hemisphere and one right hemisphere), and one patient had a single small lesion in the right corpus callosum. The number of lesion clusters in each TBI subject ranged from 1 to 14 (M = 7.1, SD = 3.42), while total lesion volume ranged from 38mm^3^ to 164,291 mm^3^ (M = 30,274.41, SD = 38,582.98). The 14 lesion profiles are shown visually in Figure 1. Moreover, lesion descriptions observed on T1w MRIs are provided by a neuroradiologist (PB) (Table 1).

**Table 1:** An overview of the demographic and clinical variables of the 14 individuals with ms-TBI.

TBI ID	Sex	Age years	TSI years	Cause	Lesion Vol mm^3^	Lesion clusters	Description of lesions at the time of scanning for original research study
TBI01	M	37	3.92	Bicycle accident	76,645	7	Severe B inf. and ant. FL EM (R) is worse than (L). Moderate B ant. Temp. EM. Small area of (L) parieto-occipital. EM. (R) PL ventricular drain tract.
TBI02	M	30	29	Pedestrian motor vehicle accident	67,270	10	Severe B ant. and med. Frontal. EM involving the ant. CC. Small area of (R) middle frontal. EM. Moderate (R) PL EM. Small focal T1-w hypo (L) frontoparietal WM. Focal T1-w hypointensity in the body of the CC.
TBI03	M	60	5	Motor vehicle accident	38	1	Small focal T1-w hypointensity in the body of the CC.
TBI04	M	45	21	Vehicle accident	819	1	Small EM in the (R) precentral gyrus
TBI05	M	49	15	Vehicle accident	164,291	4	Severe EM involving both ant. and inf. FL, (R) TL, and (R) parietotemporal region extending to the (R) post. FL. Focal T1-w hypointensity in the anteromedial aspect of the (L) thalamus. Volume loss and T1-w hypointensity on the ant. Body and genu of the CC
TBI06	F	49	3	Fall	17,762	7	B ant. and inf.frontal EM, (R) greater than (L) and (R) ant. Temp. EM. Small focal T1 hypointensity in the ant. Body of CC.
TBI07	F	29	15	Fall	17,713	4	B inf. F and (L) ant. Temp. EM. Small area of EM (L) superior frontal gyrus. (R) F burr hole with underlying ventricular drain tract.
TBI08	M	17.5	2.24	Ski accident	10,078	9	Moderate EM B inf. FL. Focal T1-w hypointensity and volume loss post. Body of CC and focal T1 hypointensity genu of the CC. Small focal EM (L) PL and (L) cerebellum. Small focal T1-w hypointensity (L) PL WM.
TBI09	F	17	2.8	Traffic Accident	17,676	7	Small volume (R) orbitofrontal and B temp. EM.
TBI10	F	17.5	1.37	Horse Accident	18,150	5	Moderate B Ant. FL, (R) ant. Temporal. and B frontoparietal EM. (R) F ventricular drain tract.
TBI11	F	15.5	3.15	Traffic Accident	22,908	8	Moderate B Inf. F, (R) sup. Frontal and (L) ant. Temporal. EM. Two (R) F ventricular drain tracts. Masked artifact.
TBI12	F	14.5	1.29	Traffic Accident	27,832	12	Moderate (L) inf., ant. and middle F EM. Moderate ant. Sup. (L) temp. EM. Small focal T1 hypointensity (R) FL WM. Moderate linear T1-w hypointensity (R) PL WM. Small (R) orbitofrontal EM. Small T1-w hypointensity (R) cerebellum.
TBI13	F	11	1.2	Traffic Accident	3,720	13	Small volume (L) ant. Lat. EM.
TBI14	M	15.6	NA	NA	5,906	4	Small volume B ant. FL and (L) temp. EM. and small volume (R) PL EM.

M, male; F, female; yrs, years; m, months; B, bilateral; (R), right; (L), left; EM, encephalomalacia; ant., anterior; inf., inferior; temp., temporal; med., medial, FL, frontal lobe; TL, temporal lobe; PL, parietal lobe; OL, occipital lobe; CC, corpus callosum; med., medial; NA, not applicable.

### 2.2 MRI acquisition and processing

**Figure 2:**
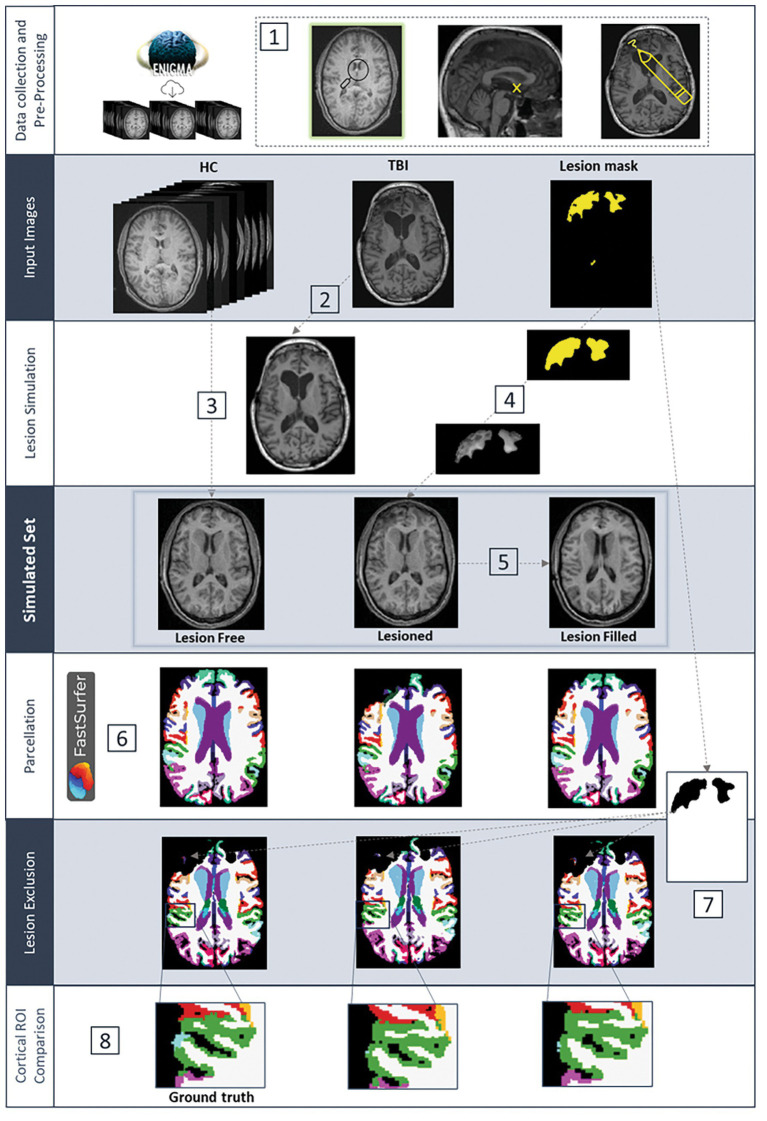
Outline of the main processing stages from data collection through to ROI Comparison. (1) Data harmonization including quality checking, setting origin to the anterior commissure and lesion delineation; (2) initial VBG filling of the native TBI T1-w scan; (3) warping of the healthy control (HC) images to the initial filled brain to produce the “*lesion-free*” images; (4) extraction of the lesion and insertion into the lesion-free images to produce the “*lesioned*” images; (5) VBG filling of the lesioned images to produce the “*VBG-filled*” images; (6) automated brain parcellation with FastSurfer’s recon-surf pipeline; (7) removal of lesioned areas from the parcellation; (8) qualitative and quantitative comparison of cortical region parcellation accuracy of the lesioned and VBG-filled images, compared to the ground truth, lesion-free parcellated images.

T1w MRI images were collated from 15 different cohorts shared by investigators within the ENIGMA adult and pediatric ms-TBI working groups. An overview of each cohort’s scanning parameters can be found in Supplementary Table 1, and the processing pipeline can be found in Figure 2. Raw T1w MRI images underwent a visual quality check in SPM12^1^ to ensure images were free from pronounced artifacts, ringing, or blurring. Four HC images were excluded at this stage, and replacement images were chosen. After orientation was uniformly set across all images, the origin was manually placed at the anterior commissure. All images were then rescaled, resulting in consistent dimensions of 256 × 256 × 256 (field of view) and a voxel size of 1 mm^3^ across the images. HC images underwent an initial whole-brain parcellation (Desikan–Killiany–Tourville (DKT) atlas) using FastSurfer’s *recon-surf* pipeline (v1.0.0, e4ed6f7) and were subsequently visually inspected by ED. No major errors were identified, enabling all HC images to be included.

### 2.3 Lesion delineation

All lesion masks were drawn in T1w native space using FSLeyes version 0.27.3 in FSL version 6.0.1.^2^ Lesions were delineated by ED, who was trained in lesion identification by neuroradiologist PB, with masks subsequently reviewed by PB. Delineation followed an in-house systematic search method and lesion identification protocol (37), based on previous protocols using T1w MRI scans only (38, 39). Abnormalities resulting in tissue loss, such as regions of encephalomalacia and damage from surgical drainage tracts, were included, while enlarged ventricles were not. The mask included non-lesion MRI abnormalities such as hyperintensities occurring in proximity to the skull (e.g., from surgical craniotomy clips), because they are known to disrupt automated algorithms during registration and segmentation (40). To reflect the inclusion of these non-lesion abnormalities, this study will refer to *repair masks* instead of lesion masks throughout the manuscript.

### 2.4 Virtual brain grafting

The VBG lesion filling workflow described in detail in Radwan et al. (25) provides two methods, one for unilateral (uVBG) and one for bilateral (bVBG) lesions. As the majority of TBI subjects in this sample had bilateral lesions, and the bVBG workflow exhibits greater accuracy in unilateral lesions compared to uVBG, we chose to run bVBG (25). HD-BET is the name of a brain extraction tool, an expanded version of the name was not provided in the original paper introducing the tool (41), before undergoing brain extraction using HD-BET (42). The pre-processed input brain image is then warped to VBG’s single-subject normative template brain in MNI space using cost-function masking. Following this, the repair mask is subtracted from the brain mask before undergoing segmentation to produce tissue probability maps (TPMs) (43).

The second step in bVBG involves flipping the pre-processed, warped input brain image along the right–left axis and performing further iterative deformation to match the template brain (41). Next, an initial donor brain image (a TPM-based T1w image) is created by combining the inverse warped template, prior TPMs, and the subject’s brain image (with the *repair mask* excluded). In bVBG, this synthetic brain is used to derive the initial filled brain, which is then segmented with Atropos (43).

In the final step of lesion filling, the initially filled brain is warped back to native space and sharpened with ANTs (41) to create the final donor brain image. Using a 2-mm FWHM smoothed and dilated mask, the *repair mask* is subtracted from the initial filled brain image before being inserted into the recipient image. To generate a realistic *lesion-free* whole head T1w image, the skull and noise are added back in, and the initial transformation is reversed to re-generate the image in native space. In this study, dilation of the mask was not performed to ensure the regions outside of the mask were unaffected by any processes of VBG.

### 2.5 Lesion simulation

Lesion simulation was performed using the same steps as described in a previous study (25) (see Figure 2). Using one HC image, one TBI image, and the *repair mask* as input, lesion simulation generates three images: (1) a “*lesion-free*” image (created by registering the HC to the TBI native image space), which acts as a ground truth; (2) a “*lesioned”* image (created by inserting the TBI lesion onto the *lesion-free* image); and (3) a “*VBG-filled”* image (created by performing VBG lesion filling on the *lesioned* image). Each set of three images—*lesion-free, lesioned,* and *VBG-filled*—created from the same input images is identical in all regions outside of the lesion, enabling FastSurfer parcellations from the *lesion-free* images to be used as the ground truth for qualitative and quantitative analysis.

TBI images and their corresponding binary repair masks (all in native space) were repaired by VBG in TBI native space. The *lesion-free* images were generated using ANTs (41) non-linear warping of each set of 10 HC T1w brain images onto their matched repaired TBI image. These *lesion-free* images can resemble increases in general atrophy or enlarged ventricles commonly associated with TBI (44) but are not influenced by the presence of a lesion.

The *lesioned* images were created by matching the intensity histogram of the original TBI T1w brain image to the *lesion-free* images. The repair masks were smoothed (2-mm FWHM 3D Gaussian kernel) before being used to extract the histogram-matched lesioned area and insert it in the corresponding location on the *lesion-free* images. This process generates synthetically *lesioned* images that are identical to the *lesion-free* images in all regions outside of the *repair mask.*

To generate the *VBG-filled* images, the *lesioned* images were processed using bVBG (25). After lesion simulation, the *VBG-filled* images were observed to have visible global textural differences and were then identified to have intensity histograms noticeably different from the *lesioned* and *lesion-free* images. All *VBG-filled* images therefore underwent intensity matching using MRtrix3 (45) *mrhistmatch-scale* using the *lesion-free* ground truth images as the reference image (see Supplementary materials for details of histogram matching and intensity histogram plots, Supplementary Figure 1).

### 2.6 Cortical parcellation

Cortical parcellation of all three groups of brain images, *lesion-free* (*N* = 140), *lesioned* (*N* = 140), and *VBG-filled* (*N* = 140), was conducted using FastSurfer’s (v1.0.0, e4ed6f7) *recon-surf* pipeline (20). In line with previous studies (17, 18) we selected 62 cortical regions (31 pairs across hemispheres) of interest (ROIs) from the Desikan–Killiany–Tourville atlas (46). Each *recon-surf* run was allocated 2.5 h of runtime. Runs that quit with an error unrelated to time were considered to have failed. Each image was provided two attempts to achieve successful parcellation, with run time and computing allocations kept consistent across both runs. Portions of the parcellation that fell within the *repair mask* were removed from the *lesion-free*, *lesioned,* and *VBG-filled* images.

### 2.7 Spatial alignment

To compare the spatial accuracy of FastSurfer’s parcellations, ANTs *DiceandMinDistSum* (8) was used to calculate the Dice similarity coefficient (DSC) for each cortical ROI in the *lesioned* and *VBG-filled* images, relative to that same ROI in the *lesion-free* images. DSC is a measure of spatial similarity between two images or regions, ranging from 0 (no similarity) to 1 (perfect overlap). The calculation of DSC for the *VBG-filled* image is expressed in formula 1 (where *VBG-filled* is substituted with *lesioned* as needed):


$$\mathrm{DSC}=2\times \frac{\left(\text{lesion}\_\text{free}\_\mathrm{ROI}\cap \mathrm{VBG}\_\text{filled}\_\mathrm{ROI}\right)}{\left(\text{lesion}\_\text{free}\_\mathrm{ROI}+\mathrm{VBG}\_\text{filled}\_\mathrm{ROI}\right)}$$

### 2.8 Volume

Volume of each cortical ROI in all images was calculated using ANTs *LabelOverlapMeasures* (8). The percent volume difference (PVD) was calculated by comparing the ROI volume of the *lesioned* and *VBG-filled* images to that same ROI volume in the ground truth *lesion-free* images. This can be expressed in formula 2 for the *VBG-filled* image (which can be substituted by the *lesioned* image as needed):

$$\mathrm{PVD}=\frac{\left(\mathrm{VBG}\_\text{Filled}\_\mathrm{ROI}\_\mathrm{Vol}-\text{Lesion}\_\text{Free}\_\mathrm{ROI}\_\mathrm{Vol}\right)}{\text{Lesion}\_\text{Free}\_\mathrm{ROI}\_\mathrm{Vol}}\times 100$$

Images with a mean percent volume difference score closer to zero have volumes more similar to the *ground truth* compared to those with larger values. Negative values indicate that *lesioned* and *VBG-filled* images are smaller than the ground truth volumes, while positive values indicate volumes greater than the ground truth.

### 2.9 Statistical analysis

All statistical analyses were performed in R version 4.2.2 (2022-10-31 ucrt), within RStudio 2022.12.0 + 353. To compare the parcellation accuracy of *lesioned* and *VBG-filled* images with respect to the ground truth images, the DSC and PVD were calculated for all cortical regions and then averaged to generate a mean DSC and PVD value for each individual image. Paired samples *t*-tests were then used to investigate group differences between the mean DSC and PVD values for the *lesioned* and *VBG-filled* images when compared to the *lesion-free* images. An exploratory sensitivity analysis was conducted using Spearman correlations to investigate the impact of lesion volume and intensity distribution RMSE on parcellation accuracy (DSC and PVD) for image type, and the association between lesion volume and RMSE for image type. All results were considered significant at a *p* ≤ 0.05.

## 3 Results

### 3.1 Qualitative assessment

After visual inspection of the lesion simulation, the *lesioned* images generated from TBI03 and TBI13 were not successfully simulated (Supplementary Figure 2 and Supplementary Table 2 for examples). Therefore, all simulated sets of images generated from TBI03 and TBI13 were removed from subsequent analysis.

The remaining 360 brain images were run through the FastSurfer *recon-surf* pipeline. The majority of images (*N* = 350) achieved successful FastSurfer parcellation on the first attempt. Of the images generated from TBI05 (largest total lesion volume, 16,4291 mm^3^), FastSurfer failed to successfully complete parcellation in four of the *VBG-filled* images and six of the *lesioned* images. After a second attempt, all four *VBG-filled* images completed parcellation successfully, while parcellation for the six *lesioned* images failed once again. Those six *lesioned* images, along with their corresponding *lesion-free* and *VBG-filled* images, were excluded from group comparisons. The final analysis included 114 simulated sets with 342 individual images.

**Figure 3:**
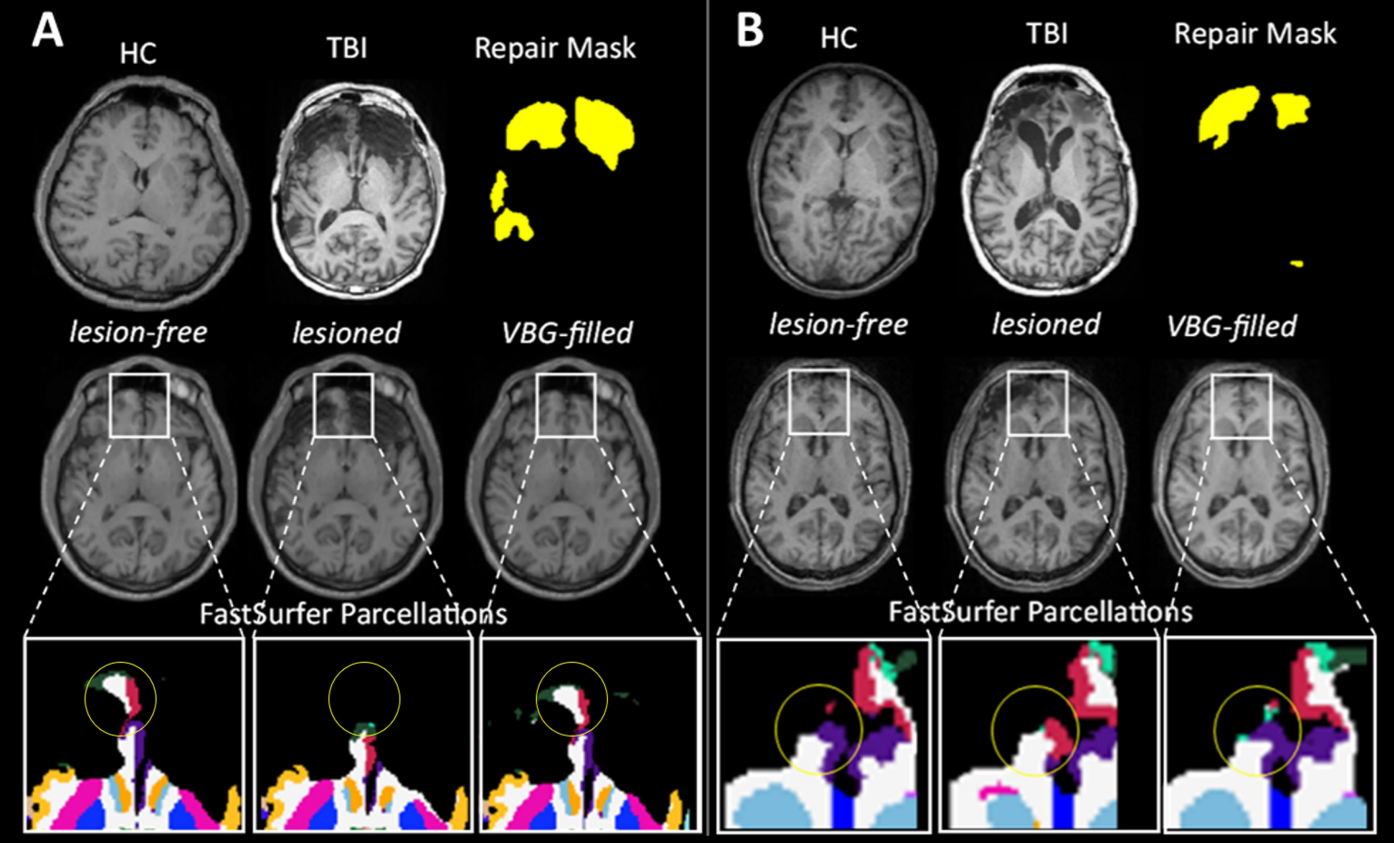
Visual examples of the original input images, simulated set of images, and the resultant parcellations for four lesion profiles. **(A)** on the left depicts TBI05 and **(B)** on the right shows TBI01. The top row depicts the input images for lesion simulation; the original healthy control (HC) image, and the raw ms-TBI image (TBI) with the corresponding binary lesion mask. The middle row depicts the three output images from lesion simulation; the *lesion-free*, *lesioned,* and *VBG-filled* images. In the bottom row, zoomed-in sections of the cortical parcellation are shown with the yellow circles highlighting regions where the *VBG-filled* parcellations appear visibly more similar to the *lesion-free* ground truth compared to the *lesioned* parcellations.

Visual inspection of VBG lesion filling revealed predominantly anatomically plausible filling. WM lesions appeared to be filled more coherently, with smooth boundaries making them almost undetectable without prior knowledge of their location, while the filling of cortical gray matter exhibited more distinct boundaries and visible textural differences (Supplementary Table 3). Inspection of the raw FastSurfer parcellation of *lesioned* images revealed misclassification errors where, when cortical regions were missing due to the presence of a large lesion, the missing tissue had been incorrectly assigned to nearby normal-appearing tissue (Supplementary Table 1 and Supplementary Figure 3). Figure 3 depicts examples where, in the regions neighboring a lesion, parcellations produced after VBG filling were in line with our expectations, appearing visually closer to the *lesion-free* ground truth images compared to the *lesioned* images.

### 3.2 Quantitative assessment

Paired-samples *t*-tests were conducted to compare the mean Dice similarity coefficient (DSC) and mean percent volume difference (PVD) scores between *lesioned* (DSC M = 0.93, SD = 0.03; PVD M = −0.40, SD = 1.7) and *VBG*-*filled* images (DSC M = 0.81, SD = 0.07; PVD M = −9.03, SD = 7.72). Checks for the assumption of normality identified two images as statistical outliers, as their values were beyond 1.5 times the interquartile range from the quartiles. These two images and their corresponding images in that simulated set were excluded.

**Figure 4:**
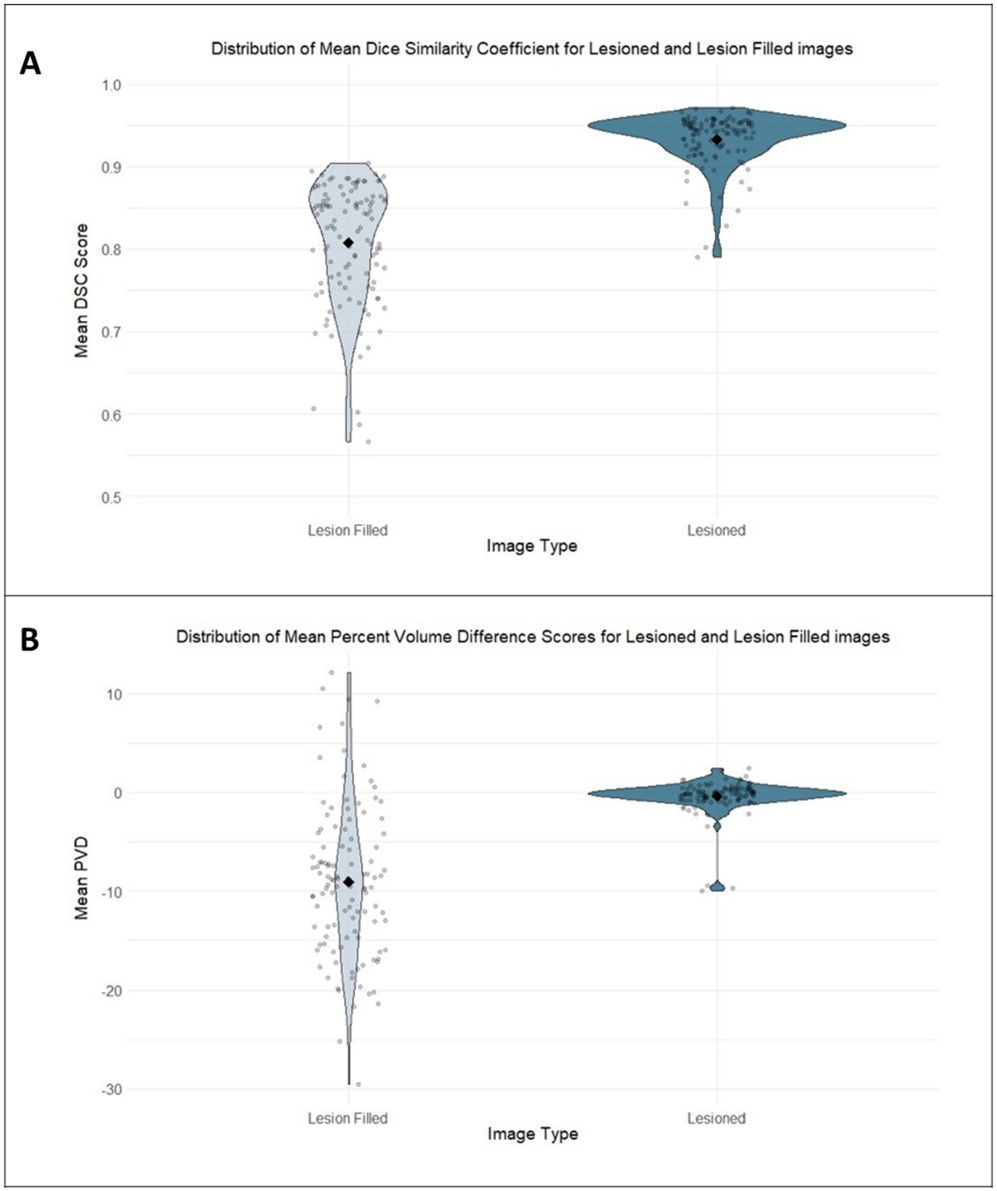
**(A)** Distribution of mean DSC for lesioned and VBG-filled images. **(B)** Distribution of mean PVD for lesioned and VBG-filled images. Violin plots show the distribution of mean DSC **(A)** and PVD scores **(B)** for the *lesioned* and *VBG-filled* images, calculated with respect to the ground truth *lesion-free* images. **(A)** The mean DSC scores for the *VBG-filled* images are lower than those for the *lesioned* images, indicating that *lesioned* images were more closely spatially aligned with the ground truth. **(B)** The narrower distribution of PVD scores clustered approximately zero for the *lesioned* images shows less volumetric difference (indicating more accurate parcellations) than the broad distribution of *VBG-filled* images when compared to the ground truth.

A paired-samples *t*-test comparing the spatial similarity of *lesioned* and *VBG-filled* images relative to the ground truth revealed a statistically significant difference in DSC between image types [*t*(111) = 19.5, *p <* 0.001, *d* = 1.49 (SD = 0.08)]. Specifically, the *lesioned* images produced a higher dice score (95% CI [0.11, 0.14]) compared to the *VBG-filled* parcellation images. In other words, the parcellations of the *lesioned* images were more closely aligned with the ground truth images (i.e., *lesion-free* parcellations) than the parcellations of the *lesion-filled* images (see Figure 4A).

A paired-samples *t*-test comparing mean percent volume difference (PVD) of the *lesioned* and *VBG-filled* images relative to the ground truth volumes revealed a significant difference in PVD between image types [*t*(111) = 11.3, *p <* 0.001, *d* = 1.22 (SD = 7.07)]. Specifically, the average PVD was 5.08% (95% CI [7.12, 10.1]) lower in the *lesioned* compared to the *VBG-filled* images. In respect to the ground truth parcellations, images that had undergone VBG lesion filling exhibited a significant estimation of regional cortical volumes when compared to *lesioned* images that had not undergone filling. Taken together, the *lesioned* images showed both a higher spatial similarity with and a lower PVD from the ground truth *lesion-free* images compared to the *VBG-filled* images (see Figure 4B).

An exploratory sensitivity analysis conducting Spearman correlations in the l*esioned* images revealed significant negative associations between lesion volume and both DSC [*ρ*(112) = −0.56, *p <* 0.001] and PVD [*ρ*(112) = −0.33, *p <* 0.001], suggesting that the parcellation accuracy in these images decreased as lesion volume increased. In contrast, no significant correlations were observed for *VBG-filled* images and parcellation accuracy (DSC: *ρ*(112) = 0.14, *p* = 0.15; PVD: *ρ*(112) = 0.17, *p* = 0.078). With respect to RMSE, *lesioned* images showed a significant negative association with both DSC [*ρ*(114) = −0.62, *p <* 0.001] and PVD [*ρ*(114) = −0.36, *p <* 0.001]. Similarly, for *VBG-filled* images, RMSE was strongly negatively correlated with DSC [*ρ*(114) = −0.81, *p <* 0.001] and PVD [*ρ*(114) = −0.22, *p* = 0.020]. These findings indicate that as histogram misalignment (higher RMSE) increased, parcellation accuracy decreased. Finally, investigating the relationship between lesion volume and RMSE showed that increasing lesion volume was associated with higher RMSE between intensity distributions for lesioned images relative to lesion-free images (*ρ* = 0.88, *p <* 0.001, *n* = 114). By contrast, RMSE for VBG-filled images was weakly but significantly negatively correlated with lesion volume (*ρ* = −0.25, *p* = 0.008, *n* = 114), suggesting that histogram similarity to lesion-free images improved slightly with larger lesion sizes (see Supplementary Figures 4–8).

## 4 Discussion

This lesion simulation study investigated, for the first time, the utility of VBG lesion filling alongside FastSurfer parcellation in ms-TBI patients. Qualitative inspection of both VBG inpainting and the corresponding image intensity distributions was complemented by a direct quantitative comparison of spatial (DSC) and volumetric (PVD) similarity metrics between *lesioned* and *VBG-filled* parcellation images compared to the *lesion-free* ground truth.

Qualitative observations were consistent with previous findings (25, 32). VBG enabled successful completion of FastSurfer whole-brain parcellation of six images with the largest focal lesions, whereas, without it, the corresponding *lesioned* subjects failed to successfully complete parcellation. Close visual inspection revealed that VBG’s lesion filling achieved more realistic results and smoother boundaries for WM lesions, while at the edges of the cortical ribbon, the filled regions exhibited visibly distinct boundaries and noticeable textural differences. The distinct boundaries observed in our study could have been influenced by deviations from the default bVBG pipeline.

In this study, VBG’s optional site-specific template creation was not appropriate due to the aggregation of images across multiple sites. Additionally, during our piloting of VBG, we observed that dilating the lesion mask resulted in image alterations outside the original masked area. Therefore, in the final pipeline, we opted against lesion mask dilation to preserve the integrity of healthy tissue for subsequent analyses. Mask dilation inevitably incorporates healthy tissue into the inpainting process, requiring subsequent exclusion of these regions from downstream analyses. It is possible that smaller lesions in our sample may have benefited from lesion mask dilation to improve boundary continuity and potentially reduce locally induced parcellation errors. However, for patients with the majority of extensive lesions, preserving remaining healthy tissue may be worth prioritizing over potential boundary smoothing benefits. Assessing the trade-offs of lesion mask dilation in images with varying lesion volumes warrants future investigation.

Further qualitative inspection of image intensity histograms revealed that the VBG filling procedure introduced noticeable global shifts in intensity distributions compared to the intensity histograms of both *lesioned* and *lesion-free* images. While *lesioned* images showed intensity abnormalities consistent with lesion-induced disruptions, the *VBG-filled* images’ intensity histograms were noticeably more different from the lesion-free ground truth, manifesting as visible global textural differences in the T1w images. Interestingly, the intensity distribution differences decreased as lesion volume increased, suggesting closer alignment of *VBG-filled* image intensity distributions in images with larger lesions.

Our main hypothesis was that the *VBG-filled* images would show higher spatial similarity and lower volumetric differences to the *lesion-free* ground truth images when compared to *lesioned* images. In contrast to the original VBG study (25), this hypothesis was not supported. Parcellations from the *lesioned* images had closer spatial alignment and volumes in greater agreement with the *lesion-free* (ground truth) images when compared to *VBG-filled* images. Exploratory sensitivity analyses provided important insights into the relationship between lesion characteristics and parcellation accuracy. Larger lesion volume was significantly associated with both higher RMSE and lower parcellation accuracy in lesioned images. This finding aligns with previous literature demonstrating that larger lesions create more substantial processing challenges (25, 47). In contrast, however, the parcellation accuracy in *VBG-filled* images showed no significant association with lesion volume in this sample. Interestingly, RMSE in *VBG-filled* images was negatively correlated with lesion volume, indicating that for *VBG-filled* images, the degree of histogram misalignment reduced as lesion volume increased. This finding suggests that VBG lesion filling did provide some error reduction or improvement in the context of extensive lesion burden, but this improvement was obscured by the systematic global intensity shift introduced by VBG. We propose that this global intensity shift likely influenced FastSurfer’s cortical surface detection, resulting in a boundary shift during parcellation (see Supplementary Figure 3) (20). This provides a plausible explanation for our main finding of the reduced spatial alignment and significant underestimation of cortical volume in VBG-filled images. Taken together, these findings highlight the complexity and importance of developing and validating lesion filling methods in ms-TBI, where the extent of lesion-induced error can vary substantially between images.

### 4.1 Limitations

The results of this work must be carefully considered with respect to the limitations of this study. First, our sample composition, together with methodological aspects of the lesion simulation design, may have reduced the presence of lesion-induced error in the *lesioned* images. Specifically, the final sample contained a higher proportion of smaller lesions (<25 cm^3^) relative to larger lesions (>50 cm^3^), with a mean lesion volume of 29 cm^3^. While the range of lesion sizes included in this study is representative of ms-TBI lesions observed in other cohorts (48, 49), the relatively small number of cases with very large lesions (>86 cm^3^) limited our ability to robustly detect lesion-induced parcellation errors and to evaluate whether VBG might yield measurable improvements under conditions of extensive lesion burden. In addition, it is worth considering whether the lesion simulation procedure, involving multiple processing steps to extract and smoothly reinsert lesions, could have resulted in simulated abnormalities with lower signal disruption than those observed in native ms-TBI scans. Together, these factors likely reduced the overall level of lesion-induced parcellation error present in the dataset, thereby minimizing the potential improvements attributable to lesion filling (25).

Another important limitation of the present work is that the effect of lesion location (or proximity) on parcellation accuracy could not be investigated. A previous study in other clinical populations with unilateral lesions (e.g., stroke, glioma) has reported that brain regions in closer proximity to lesions are likely to exhibit lower parcellation accuracy (25). However, to the best of our knowledge, there is no validated measure of distance that can provide a metric encompassing multiple distances to lesions of varying sizes, which represents a methodological gap in the field. As observed in our qualitative analyses (see Figure 3 and Supplementary Figure 4), it is likely that VBG-filled images may have shown regional improvements in parcellations closer to the lesions, which could not be quantitatively captured in the design of this study. Developing and validating such metrics will be an important issue to address in future studies.

### 4.2 Future directions

The findings from this study point to several critical areas for future research. Future studies utilizing a much larger, statistically well-powered sample that encompasses a balanced spectrum of lesion sizes will be essential for disentangling how lesion inpainting and parcellation tools perform across multiple complex lesion characteristics (e.g., volume, location, and laterality). To expand upon the lesion simulation paradigm used in this study, we suggest that future work should use manually drawn parcellations as a gold standard ground truth.

Using manual gold standard parcellations would enable direct comparison between the accuracy of different parcellation tools such as FastSurfer, FreeSurfer, and multi-atlas label propagation with expectation–maximization (MALP-EM) (10, 20, 50). The choice of parcellation tool and corresponding atlas has been shown to significantly affect downstream analyses (51). Quantitatively assessing the strengths and limitations of several different parcellation tools with respect to performance across a variety of lesion characteristics could ultimately produce evidence-backed recommendations to guide both clinicians and researchers as to which tools are most appropriate for use in their clinical populations of interest.

Large-scale studies also hold the key to improving our understanding of the varied levels of lesion-induced errors and which inpainting tools are most effective for reducing these errors. The field of lesion inpainting is rapidly advancing, driven by the increased adoption of advanced deep learning architectures. Recent inpainting tools, such as the iterative frameworks of diffusion denoising models (DDM), have shown promising results in dealing with large lesions with complex boundaries crossing multiple tissue types (52, 53). Additionally, some automated tools are capable of performing end-to-end lesion segmentation and inpainting (54), which would improve the time-consuming process of manually segmenting lesion masks, which was a required input for VBG (25). As additional inpainting tools are developed for use in bilateral lesions, future studies will need to assess the accuracy of inpainting as measured by the reconstruction accuracy within the lesioned region (i.e., how closely aligned the inpainted region is with the corresponding ground truth region). Reconstruction accuracy has been identified as an important factor that can have impacts on downstream analyses such as functional MRI (55). Building a large-scale ms-TBI dataset could additionally support the training of inpainting tools optimized for use in TBI, a critical step toward inclusive pipelines, moving away from the exclusion of ms-TBI images due to extensive lesions and subsequent poor-quality parcellations.

### 4.3 Clinical implications

On the basis of our current findings, it is evident that researchers and clinicians need to apply caution when using VBG to fill ms-TBI lesions. Our results suggest that VBG filling is most useful for enabling successful parcellation in images with extensive lesions (~190 cm^3^), specifically in instances where FastSurfer might otherwise fail to produce a parcellation output. Importantly, any studies utilizing VBG for ms-TBI images should also process control images using the VBG pipeline to ensure consistent pre-processing steps and mitigate potential intensity distribution changes and parcellation boundary shifts across image types. This study provides promising results with respect to FastSurfer parcellation accuracy in images with smaller lesions (<20 cm^3^). Based on the results in our study, we tentatively suggest that lesion inpainting may not be required in studies with samples restricted to small lesions and employing FastSurfer parcellation. However, to ensure that local lesion-induced errors do not bias subsequent downstream analyses in such studies, the FastSurfer parcellation images should undergo a rigorous, systematic parcellation quality checking procedure optimized for lesioned images, such as ENIGMA’s advanced guide for parcellation error identification (EAGLE-I) (56).

By assessing the accuracy of both VBG and FastSurfer in ms-TBI images, this study provides a foundation for moving toward evidence-backed decision-making to ensure the automated tools used during neuroimaging analysis in ms-TBI are valid for the unique pathology present in each sample. Improving the confidence of tool selection and the efficiency and accuracy with which quantitative brain metrics are calculated could ultimately lead to the clinical use of quantitative brain measures. Single-subject profiles of pathology, for example, provide a way of showing how an individual’s brain differs from what is normally expected by using the parcellation to define regions of interest and then examining the connections between them (37). Having this kind of personalized profile could help identify subgroups of patients with similar patterns of disruption, which, in turn, may support more tailored treatment planning and clearer predictions about long-term outcomes.

## 5 Conclusion

This study highlights the ongoing need to develop automated neuroimaging processing tools that are specifically designed to deal with heterogeneous ms-TBI lesions. While FastSurfer’s advanced CNN-based parcellation approach seems to be robust to smaller cortical lesions, regional misclassifications were observed in larger lesions, and complete parcellation failures dominated the largest of lesion profiles. Accurate whole-brain parcellation is a crucial step for computing morphometric measures of atrophy and informing subsequent advanced analysis techniques. It is critical that automated parcellation tools and lesion filling techniques alike are thoroughly validated on images containing a variety of ms-TBI lesions (size, location, and laterality) to ensure accurate morphometry in this patient group. This will enable more representative TBI samples in future neuroimaging studies, which will have a positive impact across the wider TBI research field. The findings presented in this paper lay the groundwork for an automated MRI analysis pipeline that integrates lesion inpainting and whole-brain parcellation to produce clinically relevant pathology profiles in ms-TBI.

## Data Availability

The data analyzed in this study is subject to the following licenses/restrictions: it is possible that de-identified data from this study could be made available through joining the ENIGMA brain injury working group and agreeing to its Memorandum of Understanding. Requests to access these datasets should be directed to emily.dennis@hsc.utah.edu.

## Footnotes

1
https://www.fil.ion.ucl.ac.uk/spm/software/spm12/

2
https://fsl.fmrib.ox.ac.uk/fsl/fslwiki

## Glossary

ms-TBI: moderate-to-severe traumatic brain injury
HC: healthy control
ROI: region of interest
VBG: virtual brain grafting
WM: white matter
GM: gray matter
DSC: Dice similarity coefficient
PVD: percent volume difference
MRI: magnetic resonance imaging
T1w: T1-weighted magnetic resonance image
cT1w: contrast-enhanced T1-weighted image
T2w: T2-weighted magnetic resonance image
FLAIR: fluid-attenuated inversion recovery image
PD: proton density image
M: mean
SD: standard deviation
FOV: field of view
DKT: Desikan–Killiany–Tourville atlas
FWHM: full width at half maximum
TPM: tissue probability map
ANTs: advanced normalization tools
