# Supplementary material for: FastSurfer parcellation accuracy after lesion filling in moderate-to-severe traumatic brain injury
Source: Front Neurol. 2025 Dec 10;16:1652385. doi: 10.3389/fneur.2025.1652385 (PMC12727440; doi:10.3389/fneur.2025.1652385)
Supplement: Supplementary file 1 [file Data_Sheet_1.pdf]

Supplementary Materials

Table 1: Cohort details for participating sites and corresponding MRI acquisition parameters for each site.

Cohort Details			Subset of Subjects included from study		Scanner		Geometry			Contrast	
Country	Site	Study Reference	N (HC)	N (TBI)	Model	Field Strength	Voxel Dimensions (mm^3)	FOV (mm^2)	No. Slices	Repetition Time (ms)	Echo Time (ms)
United States of America	Pennsylvania State University	Roy et al., (2017)[1]	2	0	Siemens Magnetom Trio	3	1	-	-	-	-
	University of Southern California	Dennis et al., (2015)[2]	18	0	Siemens Trio (BMC)	3	-	-	-	-	-
					Siemens Trio (IMHRO)	3	-	-	-	-	-
	Kessler Foundation	Dobryakova et al., (2015)[3]	1	0	Siemens Allegra	3	0.859 x 0.859	256	-	2000	4.38
	Kennedy Krieger Institute	Stephens et al., (2017)[4]	5	0	Philips	3	-	-	-	7.99ms	3.76
	Baylor College of Medicine	McCauley et al., (2010)[5]	19	0	Philips Intera	1.5	1	256	-	15ms	4.6
		(Oni et al., 2010) [6]	10	0	Philips Intera	1.5	-	-	-	-	-
Belgium	KU Leuven	Caeyenberghs et al 2011[7]	6	0	Siemens Magnetom Trio	3	0.98 x 0.98 x 1.2	250	182	9.7	4.6
		(Drijkoningen et al., 2015)[8]	18	0	Siemens Magnetom Trio	3	1	240 x 256mm^2	160	2300	2.98
		Cayenberghs et al., (2011)[7]	1	0	Siemens Magnetom Trio	3	1	240 x 256mm^2	160	2300	2.98
	University of Gent	Verhelst et al., (2018) [9]	9	7	Siemens Magnetom Trio	3	1	256	176	1590	4.18
UK	Imperial College London	Jenkins et al., (2018) [10]	6	0	-	-	1	256	160	2300	2.98
	Hammersmith Hospital	https://brain-development.org/ixi-dataset/	41	0	Philips Intera	3	-	208 x 208	-	9.6	4.6
	Guys Hospital				Philips Intera Gyroscan	1.5	-	240	-	9.8	4.6
Australia	Australian Catholic University	Clemente et al., (2022) [11]	3	0	Siemens Skyra	3	1 x 1 x 1.5	220 x 220	208	2250	3.07
		Imms et al., (2022) [12]	1	7	Siemens Prisma	3	0.8	256	208	2100	2.22
		Total	140	14							

Note: '-' = MRI acquisition details not present in the relevant listed published papers

Intensity Matching

To address the mis-match observed in the intensity histogram of the *VBG-filled* image when compared to the identical histograms of the *lesion free* and *lesioned* images, post-hoc attempts at performing additional histogram matching were explored. Attempts at using the MRtrix3 [13] *mrhistmatch* (nonlinear option) and ANTs [14] *HistogramMatch* both moved the intensity histogram closer to the target *lesion free* histogram, however, both methods resulted in the presence of artefacts in some images. Utilising the MRTrix *mrhistmatch* linear option resulted in images free from artefacts, however the histogram was more dissimilar to the *lesion free* histogram than the original *VBG-filled* image histogram. Finally, using MRtrix3 *mrhistmatch* scale option, the resultant images were free from artefacts and the histogram was more closely aligned to the *lesion free* image histogram. Therefore, MRtrix3 *mrhistmatch* using the scale option, was applied to all *VBG- filled* images prior to undergoing FastSurfer parcellation.

Figure 1 Intensity histogram plots stratified by lesion volume

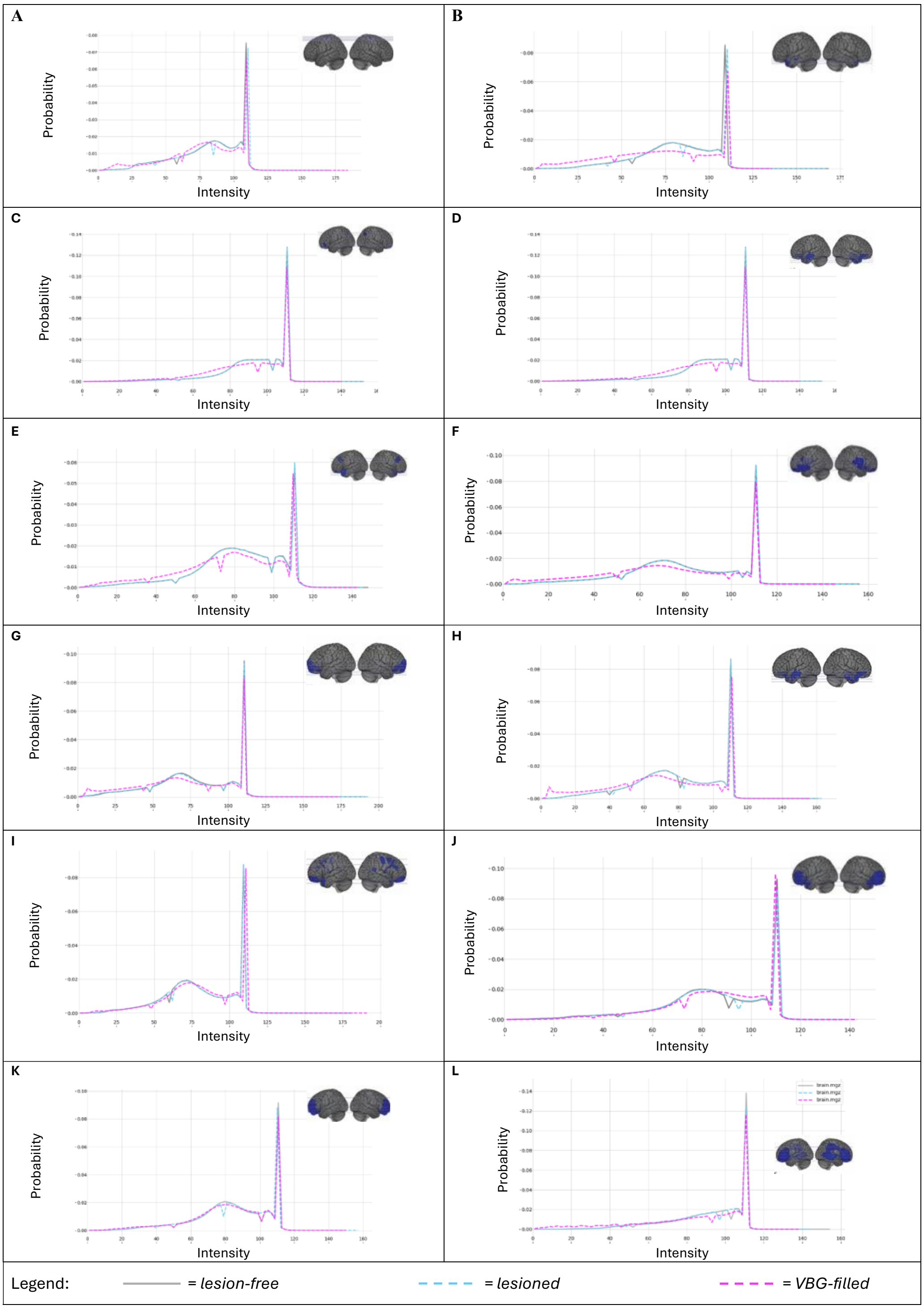

Lesion Simulation Exclusions

Two of the TBI lesion profiles included in this study were subsequently found to not have successfully produced adequate *lesioned* images during the lesion simulation process. Figure 2 below shows the zoomed in regions of the original TBI image both with and without the mask overlaid. These original images show the degree of clarity of the lesion in the original image and thus the type of lesion we expected to see after simulation. Table 2. shows two example images from these lesion profiles after simulation onto healthy control images. The red lines and circles highlight regions which visually appear no different in the *lesioned* or the *lesion free* images. The yellow lines and circles indicate where very minimal change can be noticed.

Figure 2. Original TBI image and lesion masks for the two lesion profiles excluded after lesion simulation.

TBI03 Lesion Profile

A. 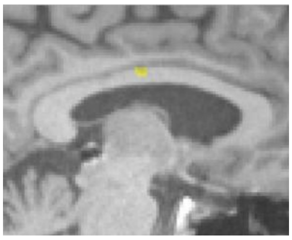

B. 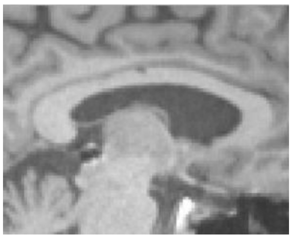

TBI13 Lesion Profile

A. 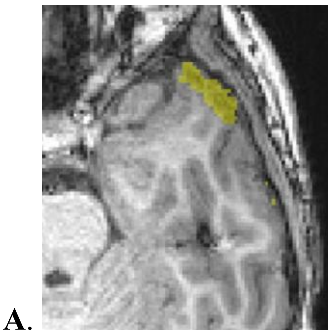

B. 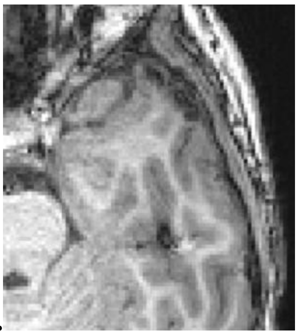

Table 2. Examples of TBI13 and TBI03 lesion profiles which did not simulate well onto the *lesioned* images and were therefore excluded from subsequent analysis.

	lesioned (with mask overlay)	*lesioned*	*lesion free* (ground truth)
TBI13 example image 1	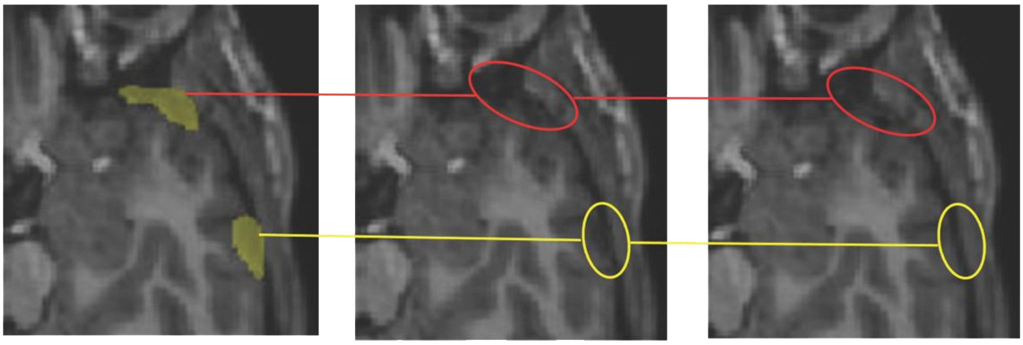		
TBI13 example image 2	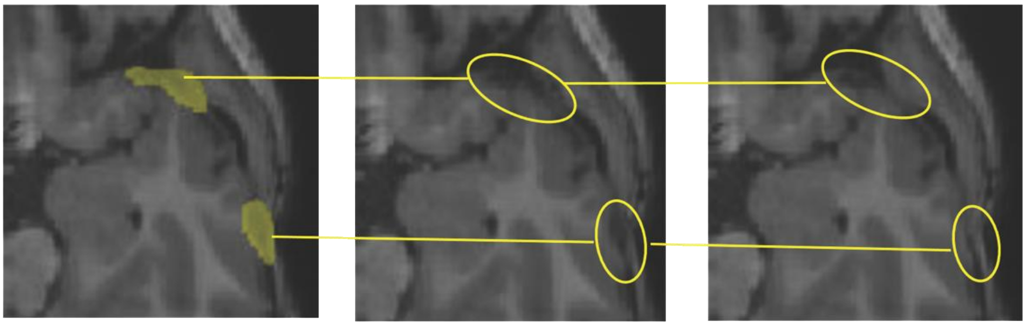		
TBI03 example image 1	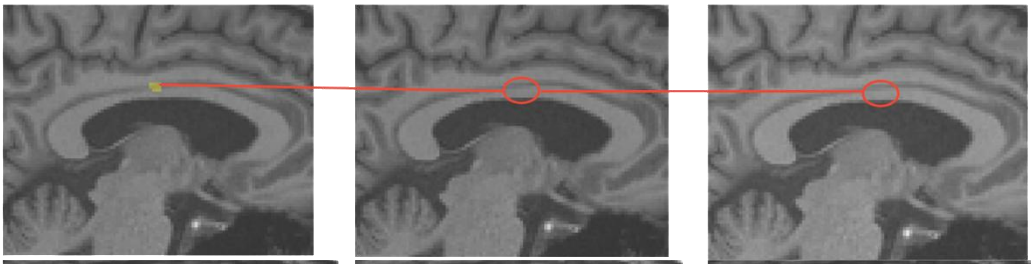		
TBI03 example image 2	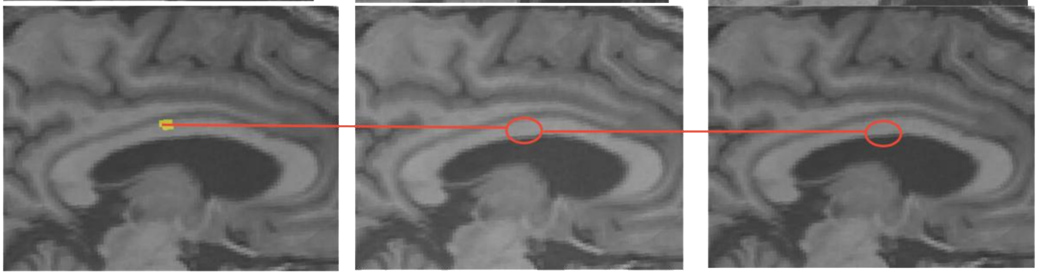		

Lesion Filling Examples

Table 3. Example lesion filling for the twelve lesion profiles included in qualitative and quantitative analysis in this study.

	lesioned	*VBG-filled* (*repair mask* overlay)	*VBG- filled*	*lesion free*
TBI01	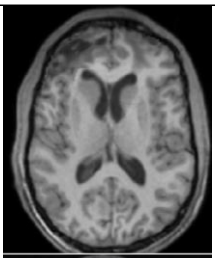	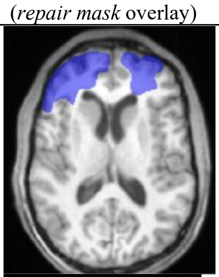	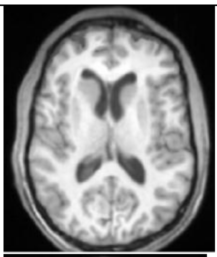	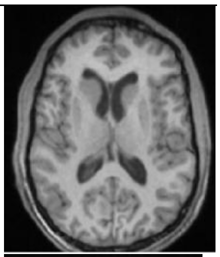
TBI02	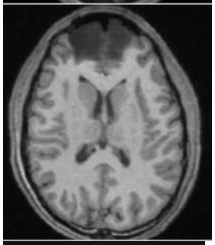	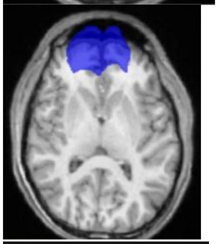	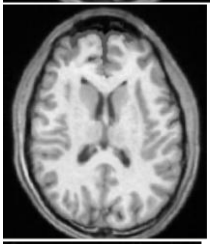	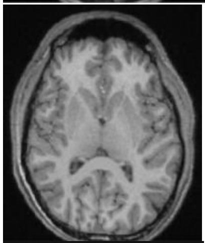
TBI04	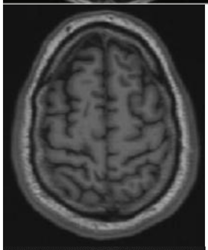	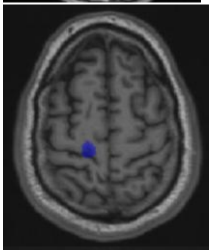	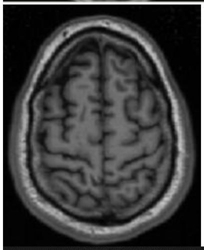	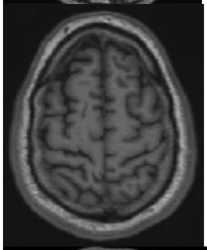
TBI05	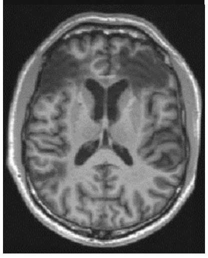	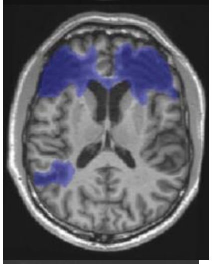	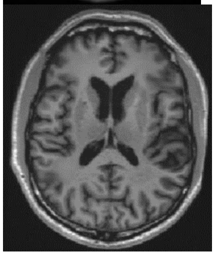	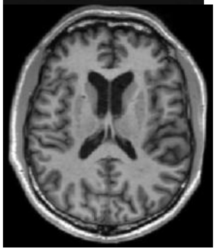
TBI06	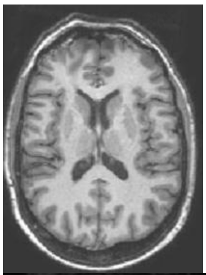	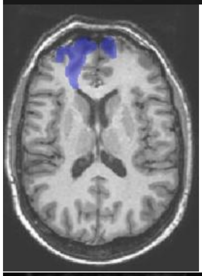	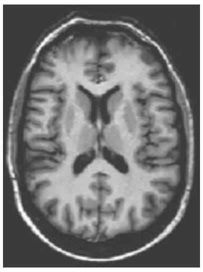	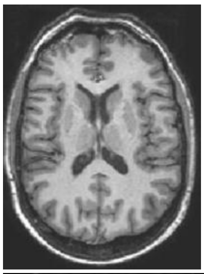
TBI07	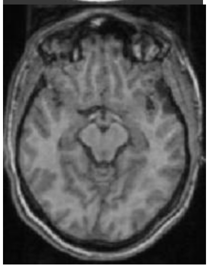	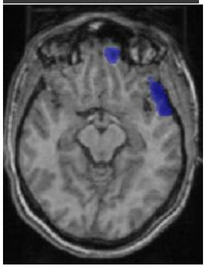	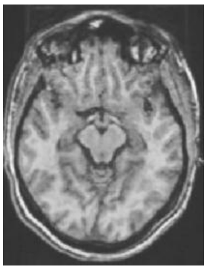	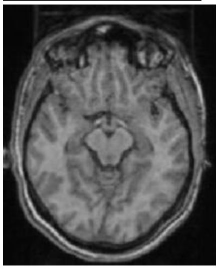

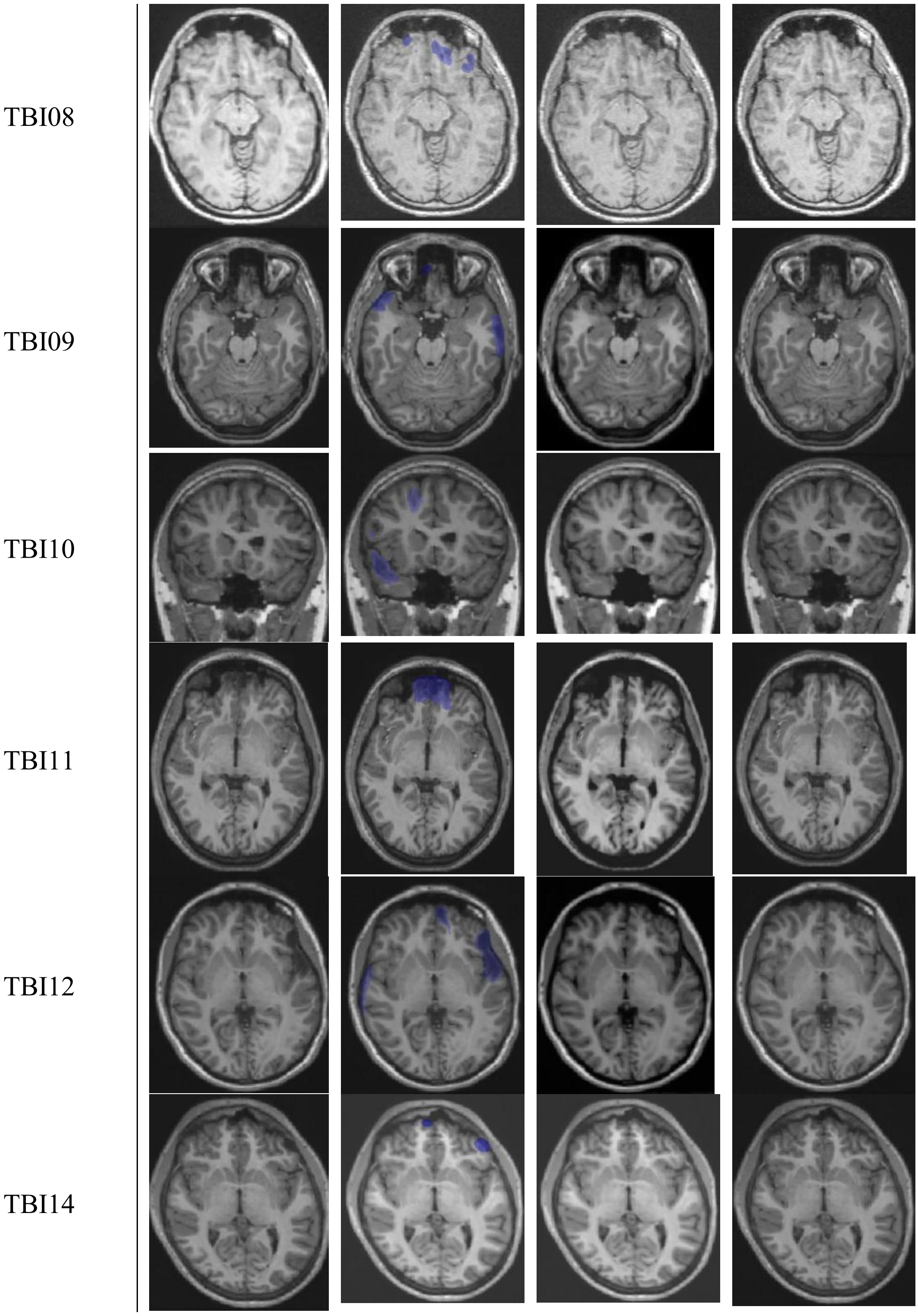
TBI08
TBI09
TBI10
TBI11
TBI12
TBI14

FastSurfer misclassification of cortical ROIs

During detailed visual inspection of the FatSurfer Parcellations it was observed that near large lesions, FastSurfer appears to be vulnerable to misclassifying portions of damaged white matter incorrectly as the cortical regions missing from within the lesioned region. Table 4 below provides four examples of images where these misclassifications are prominent.

Table 4. Examples of FastSurfer misclassification in the presence of large lesions

**TBI**	***lesioned* image**	**FastSurfer Parcellation**	**Description**
TBI01	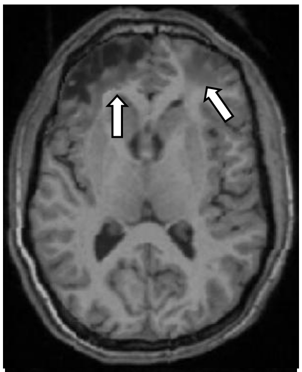	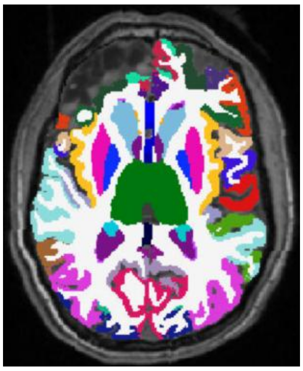	Thick white arrows on the *lesioned* image highlight areas of lesion that have been misclassified by FastSurfer and incorrectly labelled as the lateralorbitofrontal cortex
TBI02	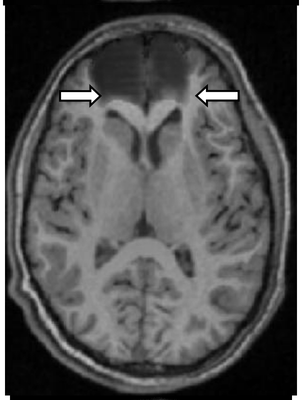	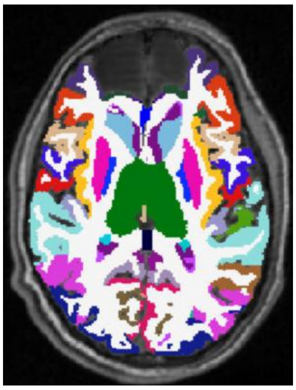	Thick white arrows on the *lesioned* image highlight areas of lesion that have been misclassified by FastSurfer and incorrectly labelled as the rostralmiddlefrontal cortex.
TBI06	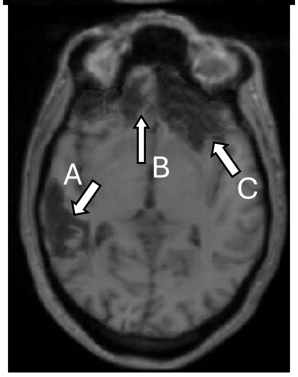	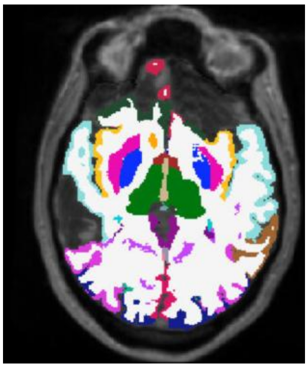	Arrow A) highlights misclassification of the right superiortemporal cortex, B) misclassification of the right lateralorbitofrontal cortex and arrow C) indicates misclassification of the left insula.
TBI12	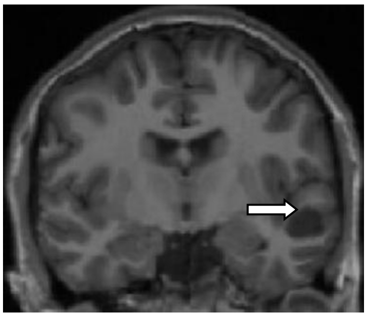	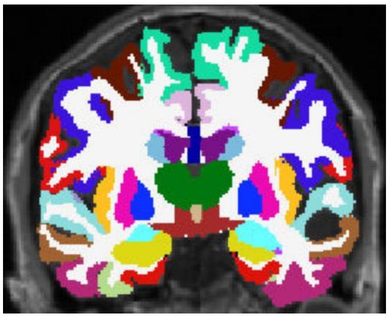	Thick white arrow on the *lesioned* image identifies the misclassification of both normal appearing and damaged white matter, both incorrectly labelled by FastSurfer as the superior temporal cortex.

Figure 3 Visual comparison of parcellation differences across image type

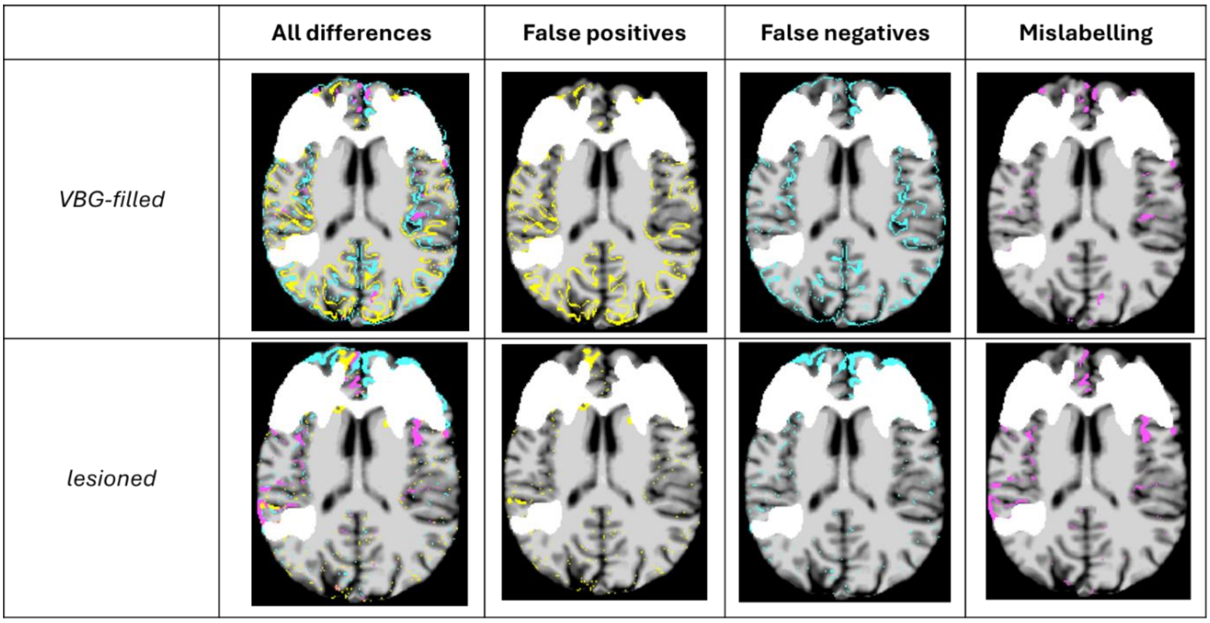

Figure 4 Scatterplot for Spearman correlation Lesion volume x DSC

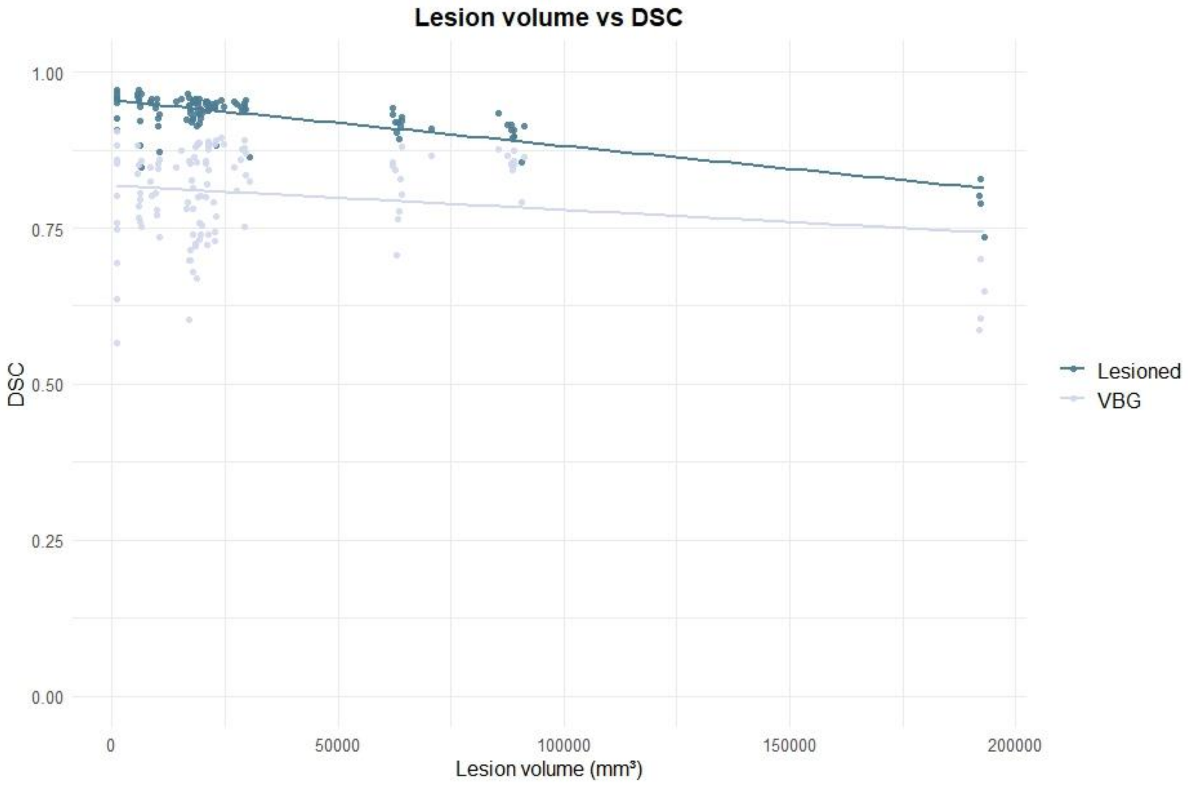

Figure 5 Scatterplot for Spearman correlation Lesion volume x PVD

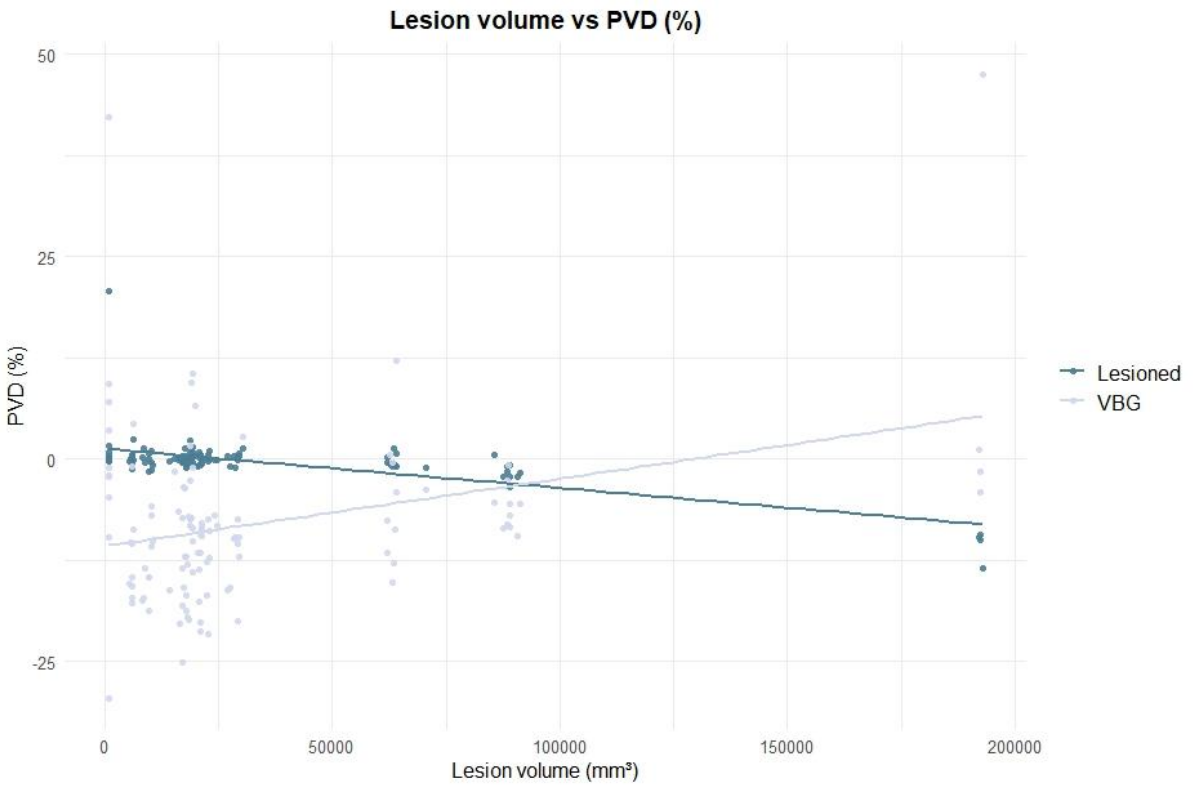

Figure 6 Scatterplot for Spearman correlation RMSE x PVD

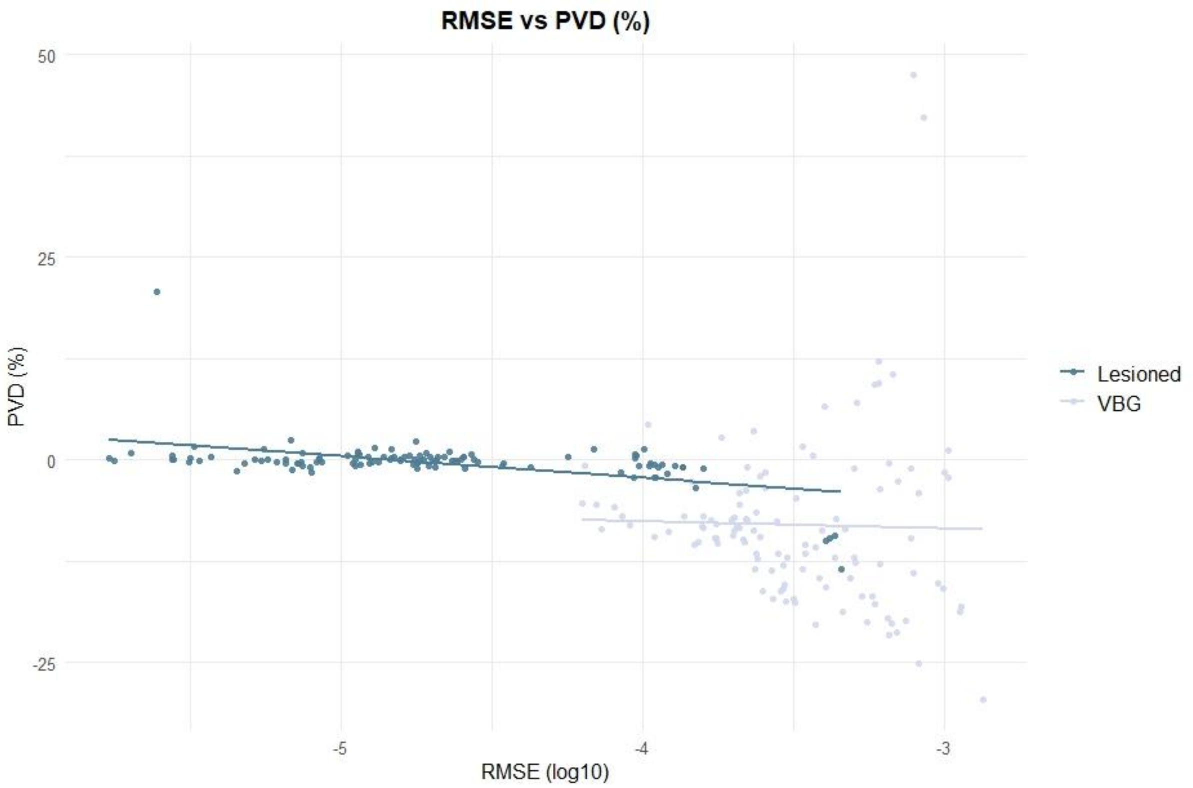

Figure 7 Scatterplot for Spearman correlation RMSE x DSC

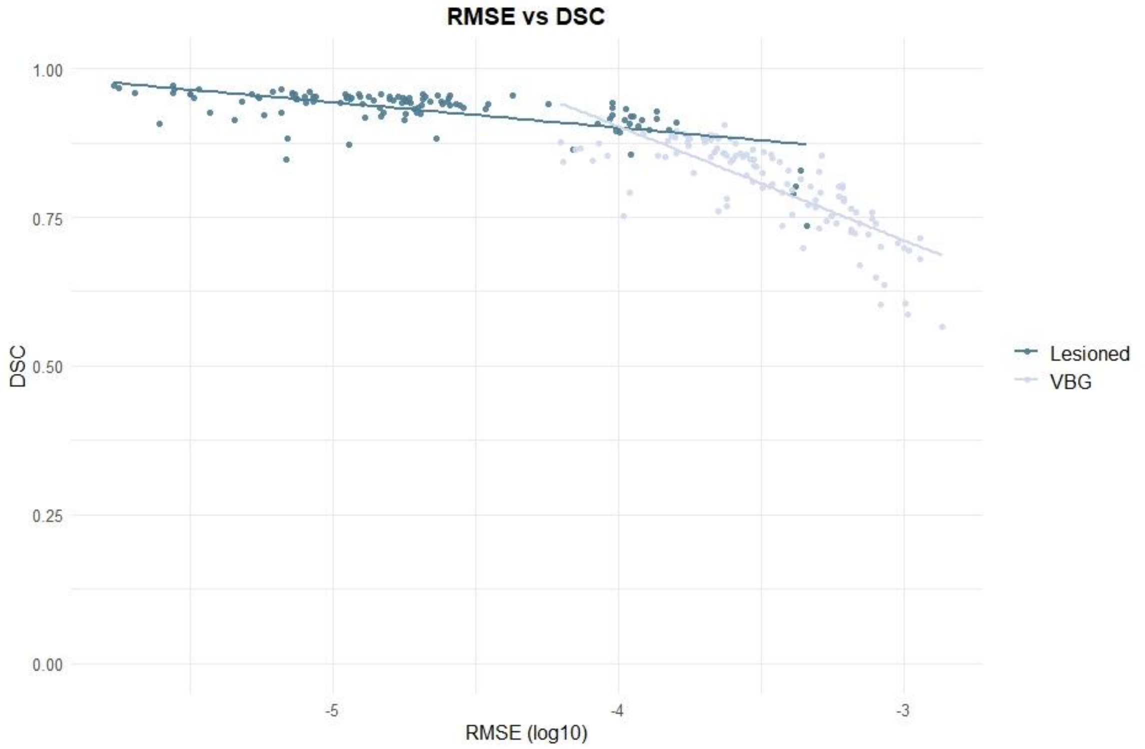

Figure 8 Scatterplot for Spearman correlation RMSE x Lesion volume

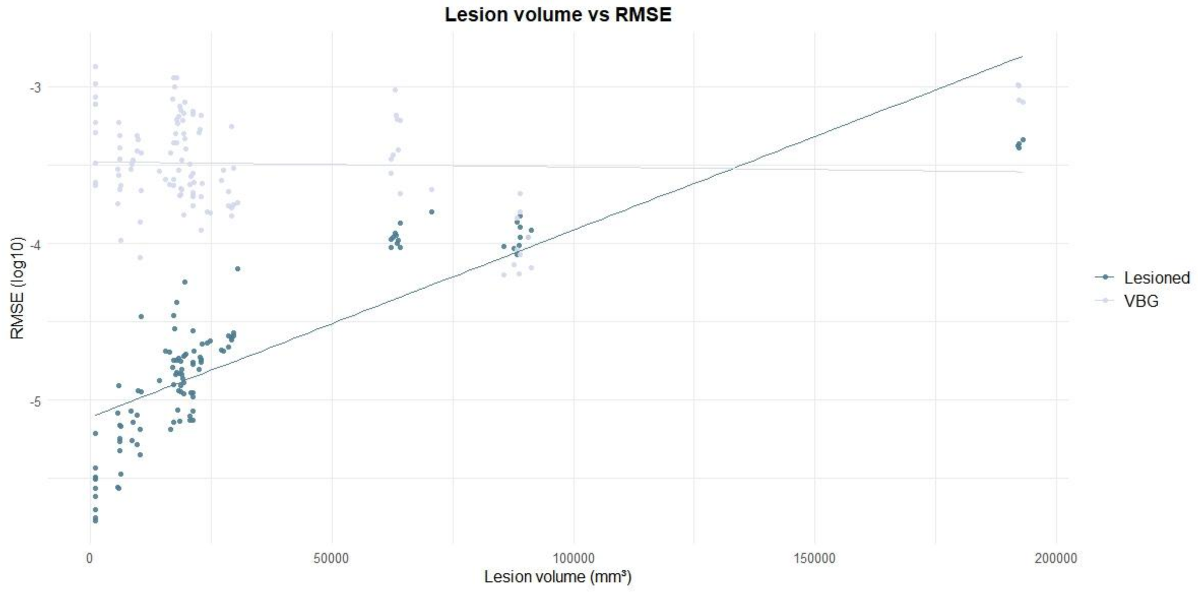

References

1. Roy A, Bernier RA, Wang J, et al. The evolution of cost-efficiency in neural networks during recovery from traumatic brain injury. PLOS ONE 2017;12(4):e0170541; doi: 10.1371/journal.pone.0170541.

2. Dennis EL, Jin Y, Villalon-Reina JE, et al. White matter disruption in moderate/severe pediatric traumatic brain injury: Advanced tract-based analyses. NeuroImage: Clinical 2015;7:493–505; doi: 10.1016/j.nicl.2015.02.002.

3. Dobryakova E, Boukrina O, Wylie GR. Investigation of Information Flow During a Novel Working Memory Task in Individuals with Traumatic Brain Injury. Brain Connectivity 2015;5(7):433–441; doi: 10.1089/brain.2014.0283.

4. Stephens J, Salorio C, Denckla M, et al. Subtle Motor Findings During Recovery from Pediatric Traumatic Brain Injury: A Preliminary Report. Journal of Motor Behavior 2017;49(1):20–26; doi: 10.1080/00222895.2016.1204267.

5. McCauley SR, Wilde EA, Merkley TL, et al. Patterns of cortical thinning in relation to event-based prospective memory performance three months after moderate to severe traumatic brain injury in children. Dev Neuropsychol 2010;35(3):318–332; doi: 10.1080/87565641003696866.

6. Oni MB, Wilde EA, Bigler ED, et al. Diffusion Tensor Imaging Analysis of Frontal Lobes in Pediatric Traumatic Brain Injury. J Child Neurol 2010;25(8):976–984; doi: 10.1177/0883073809356034.

7. Caeyenberghs K, Leemans A, Coxon J, et al. Bimanual Coordination and Corpus Callosum Microstructure in Young Adults with Traumatic Brain Injury: A Diffusion Tensor Imaging Study. Journal of Neurotrauma 2011;28(6):897–913; doi: 10.1089/neu.2010.1721.

8. Drijkoningen D, Caeyenberghs K, Leunissen I, et al. Training-induced improvements in postural control are accompanied by alterations in cerebellar white matter in brain injured patients. NeuroImage: Clinical 2015;7:240–251; doi: 10.1016/j.nicl.2014.12.006.

9. Verhelst H, Vander Linden C, De Pauw T, et al. Impaired rich club and increased local connectivity in children with traumatic brain injury: Local support for the rich? Human Brain Mapping 2018;39(7):2800–2811; doi: 10.1002/hbm.24041.

10. Jenkins PO, De Simoni S, Bourke NJ, et al. Dopaminergic abnormalities following traumatic brain injury. Brain 2018;141(3):797–810; doi: 10.1093/brain/awx357.

11. Clemente A, Attyé A, Renard F, et al. Individualised Profiling of White Matter Organisation in Moderate-to-Severe Traumatic Brain Injury Patients Using TractLearn: A Proof-of-Concept Study. medRxiv 2022;2022–03.

12. Imms P, Clemente A, Deutscher E, et al. Exploring personalised structural connectomics for moderate-to-severe traumatic brain injury. Network Neuroscience 2022;1–50.

13. Tournier J-D, Smith R, Raffelt D, et al. MRtrix3: A fast, flexible and open software framework for medical image processing and visualisation. NeuroImage 2019;202:116137; doi: 10.1016/j.neuroimage.2019.116137.

14. Avants BB, Tustison NJ, Song G, et al. A reproducible evaluation of ANTs similarity metric performance in brain image registration. Neuroimage 2011;54(3):2033–2044; doi: 10.1016/j.neuroimage.2010.09.025.
